# Rectus sheath hematoma: three case reports

**DOI:** 10.1186/1752-1947-2-22

**Published:** 2008-01-25

**Authors:** Selin Kapan, Ahmet N Turhan, Halil Alis, Mustafa U Kalayci, Sinan Hatipoglu, Hakan Yigitbas, Ersan Aygun

**Affiliations:** 1 Bakirkoy Dr. Sadi Konuk Training and Research Hospital, Department of General Surgery, Istanbul, Turkey

## Abstract

**Introduction:**

Rectus sheath hematoma is an uncommon cause of acute abdominal pain. It is an accumulation of blood in the sheath of the rectus abdominis, secondary to rupture of an epigastric vessel or muscle tear. It could occur spontaneously or after trauma. They are usually located infraumblically and often misdiagnosed as acute abdomen, inflammatory diseases or tumours of the abdomen.

**Case presentation:**

We reported three cases of rectus sheath hematoma presenting with a mass in the abdomen and diagnosed by computerized tomography. The patients recovered uneventfully after bed rest, intravenous fluid replacement, blood transfusion and analgesic treatment.

**Conclusion:**

Rectus sheath hematoma is a rarely seen pathology often misdiagnosed as acute abdomen that may lead to unnecessary laparotomies. Computerized tomography must be chosen for definitive diagnosis since ultrasonography is subject to error due to misinterpretation of the images. Main therapy is conservative management.

## Introduction

Rectus sheath hematoma is accumulation of blood in the sheath of rectus abdominis muscle due to disruption of epigastric vessels or rectus muscle [1]. Although the etiology includes trauma, abdominal operations, trocar site injury after laparoscopic operations, subcutaneous drug injections, anticoagulant therapy, hematological diseases, hypertension, coughing, physical exercise, pregnancy, it rarely occurs spontaneously [1-5]. It usually occurs in the lower quadrants of the abdominal wall and almost never crosses the midline [1,2,6]. Hematoma below the linea semicircularis causes an indirect irritation on the peritoneum due to weak posterior rectus sheath in this region leading to misdiagnoses as acute abdomen [1,2].

Three consecutive cases presenting to our Emergency Department in the past 2 months are reported in this paper regarding the accurate diagnosis and management.

## Case presentation

### Case 1

**Figure 1:**
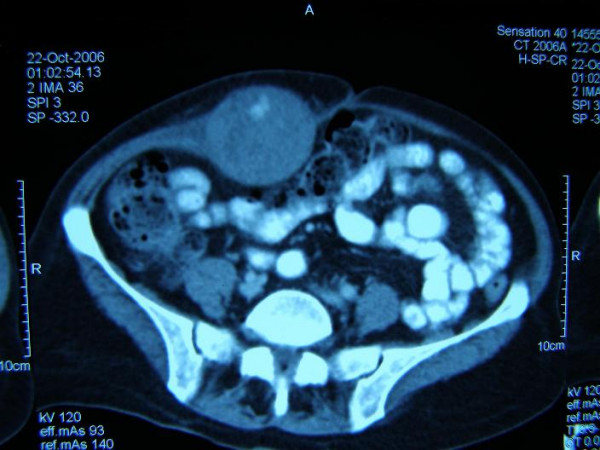
CT appearance of rectus hematoma on the right upper abdomen.

A 64 year old woman presented to the Emergency Department with complaints of abdominal pain, nausea and an abdominal mass in the right upper quadrant. She had a history of excessive coughing due to acute bronchitis. Additionally she had diabetes mellitus and hypertension under medical therapy. She had a diagnosis of Polycythemia Vera for 10 years and had been taking an antineoplastic agent containing hydroxyurea 500 mg per day since then. Physical examination revealed a large mass in the right upper quadrant of the abdomen extending to the lower abdomen. The mass was tender on palpation, but there was no rebound tenderness and muscular rigidity. Carnett and Fothergill tests were positive. Bowel auscultation revealed no pathologic sound. Mean arterial pressure was 140/80 mmHg, pulse rate was 86/min, hemoglobin level 10.56 g/dL, hematocrit 37.3%, platelet 528000/UL, activated partial tromboplastin time (aPTT) 21.8 sec, prothrombin time (PT) 17.7 sec and international normalized ratio (INR) 1.45. The other biochemical tests were normal. Ultrasound (US) examination of the abdomen confirmed a nonmobile heterogeneous mass on the right upper quadrant extending below the liver with a suspect of intestinal loop within the mass. Further imaging study of computerized tomography (CT) revealed a hematoma on the abdominal wall (Figure 1). Complete blood cell count examination on the 12th hour of admission revealed a decrease in the hemoglobin and hematocrit levels (7.63 g/dl and 22.6% respectively). Ecchymosis occurred on the third day on the abdominal wall. After intravenous fluid replacement, 3 units of erythrocyte transfusion, 2 units of fresh frozen plasma and analgesic treatment with strict bed rest, patient was discharged from the hospital on the 8th day of admission.

### Case 2

**Figure 2:**
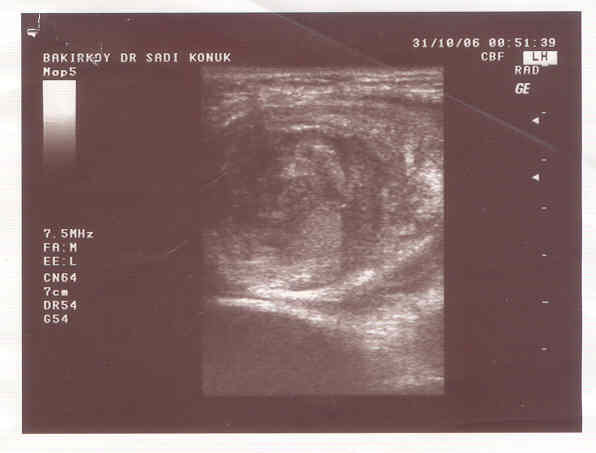
US appearance misdiagnosed as intestinal obstruction "Target sign".

A 60 year old woman presented to the Emergency Department with complaint of acute abdominal pain. She had history of chronic constipation. Physical examination revealed a painful palpable mass within the left lower quadrant of the abdomen. There was no muscular rigidity or rebound tenderness. Mean arterial pressure was 110/80 mmHg, pulse rate was 76/min, hemoglobin level 10.46 g/dL, hematocrit 32.3%, platelet 199000/UL. The other biochemical tests were normal. US revealed suspect of invaginated intestinal loop with a sign of "Target sign" (Figure 2), but the clinical examination did not confirm the prediagnosis of intestinal obstruction. CT revealed a rectus sheath hematoma. During the 3 day follow up, there was no change in the hemodynamic conditions, so no transfusion was required. Ecchymosis occurred on the third day on the abdominal wall. Partial resorption of the hematoma was observed in the follow up CT 45 days after discharge.

### Case 3

**Figure 3:**
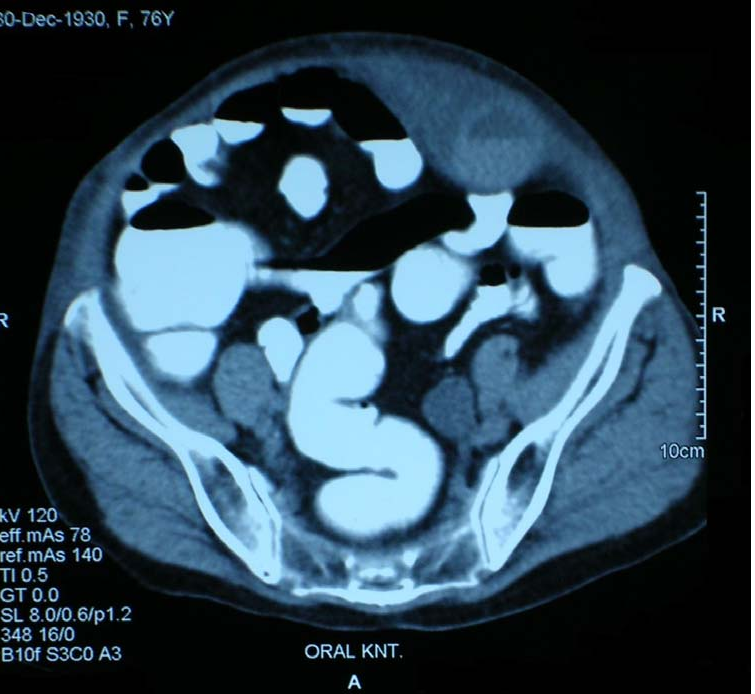
CT appearance of rectus sheath hematoma on the left side of the abdomen.

A 76 years old woman presented to the Emergency Department with complaints of abdominal pain, and an abdominal mass in the right upper quadrant. She had a history of congestive heart failure, chronic atrial fibrillation, chronic obstructive lung disease, ischemic cerebrovascular disease and myocardial infarction. The patient had been taking warfarin sodium 5 mg/day for atrial fibrillation for 2 years without medical supervision. On physical examination painful mass in the left lower quadrant and suprapubic region of the abdomen was observed. Ausculatation of the abdomen revealed hypoactive bowel sound. Mean arterial pressure was 140/80 mmHg, pulse rate 118/min, hemoglobin level 13.32 g/dL, hematocrit 30.26%, platelet 199000/UL, PT: 79.2 sec, aPTT: 56 sec, INR: 6.68. The other biochemical tests were normal. Abdominal CT revealed rectus sheath hematoma (Figure 3). During the follow up period 6 units of fresh frozen plasma was administered. Due to the hemodynamic stability patient was referred to the Internal Medicine Department.

## Discussion

Rectus sheath hematoma (RSH) is a rarely seen but important disease causing abdominal pain. There is a female predominance as may be explained by larger rectus muscle mass in man [7]. All three of our patients were also female. There are various causes resulting in RSH as abdominal trauma, previous surgery, coughing, streching, hypertension, intraabdominal injections, iatrogenic causes during laparoscopy and anticoagulation therapy [8,9]. In the abdominal wall below the arcuate line there's only transversalis fascia between peritoneum and posterior rectus sheath, therefore rupture of epigastric vessels or muscle within this sheath causes a hematoma mimicking acute abdomen [2]. Common presenting signs and syptoms are abdominal pain, abdominal wall mass, decrease in hemoglobin, abdominal wall ecchymosis, nausea, vomiting, tachycardia, peritoneal irritation, fever, abdominal distention and abdominal cramping [7].

Fothergill's sign and Carnett sign are positive in rectus sheath hematoma, and helps to differentiate this condition from intraabdominal pathologies [1,2,4,6]. Fothergill' sign is positive when the haematoma within the rectus sheath produces a mass that does not cross the midline and remains palpable when the patient tenses his rectus muscle by touching his chest using his chin [4]. Carnett sign is exacerbation of the pain and tenderness over the hematoma by contraction of rectus muscle by sitting halfway up in a supine position [2]. Both of these tests were positive in three of our patients. Echymosis on the abdomen may occur late in the follow up period. The ecchymosis may be seen in the flanks or periumblical region causing Grey Turner's and Cullen's sign [4,6].

Misdiagnosis may lead to unnecessary negative laparotomies with increase in morbidity and mortality [10]. US, CT and magnetic resonance imaging are widely used in the diagnosis. Although US seems to be the procedure of choice due to its high sensitivity rates, time and cost effectivity and low radiation in some series, however sometimes it is difficult to differentiate intraperitoneal lesions from extraperitoneal lesions by US as the technique is subject to error by means of probe induced tenderness and limitations of interpretation of the images [6]. This was the case in the first two cases as the ultrasonographers identified the hematomas as "intraperitoneal". CT is superior to US in localisation, extension and evaluation of the size of the hematoma. Moreover CT imaging can give the classification of the hematoma. According to the CT classification, Type I hematomas are mild and the hematoma occurrs within the muscle with an increas in muscle length and do not require hospitalization. Type II hematomas are moderate, the hematoma is within the muscle but bleeding occurs into the space between transversalis fascia and the muscle. Type III hematomas are severe and located between transversalis fascia and the muscle, anterior to the peritoneum and urinary bladder. Type II and III hematomas require hospitalization. In Type I hematomas hospitalisation is not usually required and the hematoma resorbs spontaneously within 30 days. In Type II lesions bed rest, intravenous fluid replacement and analgesia is the appropriate treatment. In Type III lesions additional blood product transfusions are required. These kind of hematomas resorb approximately in 3 months [4,11]. All three of our cases were Type II.

Conservative treatment is the mainstay of management in hemodynamically stable patients with non expanding hematoma [1,4,6]. In cases with failure of conservative treatment, surgical approach can be chosen but the mortality rates of surgery for rectus sheath hematoma is high. Coil embolisation can be an alternative in high risk patients refractory to conservative therapy [1,4].

## Conclusion

Rectus sheath hematoma is a rarely seen entity often misdiagnosed as acute abdomen. Prompt history taking with careful physical examination and appropriate imaging studies help the correct diagnosis avoiding unnecessary laparotomies. CT seems to be the most appropriate choice of imaging. Management is conservative in most cases including bed rest, analgesia, intravenous fluid replacement and blood transfusions when necessary.

## Competing interests

The author(s) declare that they have no competing interests.

## Authors' contributions

SK was involved in the management of the patient as well as writing the case reports. HA and MUK were involved in the management of the patients. ANT and EA has been involved in the correction of the manuscript as well as general supervision. SH and HY were involved in the design of the study and helped to draft the manuscript. All authors read and approved the final manuscript.

## Consent

Written informed consent was obtained from the patients for publication of this study.
